# Redesigning systems to improve teamwork and quality for hospitalized patients (RESET): study protocol evaluating the effect of mentored implementation to redesign clinical microsystems

**DOI:** 10.1186/s12913-019-4116-z

**Published:** 2019-05-08

**Authors:** Kevin J. O’Leary, Julie K. Johnson, Milisa Manojlovich, Jenna D. Goldstein, Jungwha Lee, Mark V. Williams

**Affiliations:** 1 Division of Hospital Medicine, Northwestern University Feinberg School of Medicine, 211 E. Ontario Street, Suite 700, Chicago, IL 60611 USA; 2 Department of Surgery and the Center for Healthcare Studies, Northwestern University Feinberg School of Medicine, Chicago, IL USA; 3 Department of Systems, Populations, and Leadership, University of Michigan School of Nursing, Ann Arbor, MI USA; 4 Center for Hospital Innovation and Improvement, Society of Hospital Medicine, Philadelphia, PA USA; 5 Department of Preventative Medicine, Northwestern University Feinberg School of Medicine, Chicago, IL USA; 6 Center for Health Services Research, University of Kentucky College of Medicine, Lexington, KY USA

**Keywords:** Interdisciplinary communication, Patient care team, Interpersonal relations, Medical errors, Hospitalization, Clinical microsystems

## Abstract

**Background:**

A number of challenges impede our ability to consistently provide high quality care to patients hospitalized with medical conditions. Teams are large, team membership continually evolves, and physicians are often spread across multiple units and floors. Moreover, patients and family members are generally poorly informed and lack opportunities to partner in decision making. Prior studies have tested interventions to redesign aspects of the care delivery system for hospitalized medical patients, but the majority have evaluated the effect of a single intervention. We believe these interventions represent complementary and mutually reinforcing components of a redesigned clinical microsystem. Our specific objective for this study is to implement a set of evidence-based complementary interventions across a range of clinical microsystems, identify factors and strategies associated with successful implementation, and evaluate the impact on quality.

**Methods:**

The RESET project uses the Advanced and Integrated MicroSystems (AIMS) interventions. The AIMS interventions consist of 1) Unit-based Physician Teams, 2) Unit Nurse-Physician Co-leadership, 3) Enhanced Interprofessional Rounds, 4) Unit-level Performance Reports, and 5) Patient Engagement Activities. Four hospital sites were chosen to receive guidance and resources as they implement the AIMS interventions. Each study site has assembled a local leadership team, consisting of a physician and nurse, and receives mentorship from a physician and nurse with experience in leading similar interventions. Primary outcomes include teamwork climate, assessed using the Safety Attitudes Questionnaire, and adverse events using the Medicare Patient Safety Monitoring System (MPSMS). RESET uses a parallel group study design and two group pretest-posttest analyses for primary outcomes. We use a multi-method approach to collect and triangulate qualitative data collected during 3 visits to study sites. We will use cross-case comparisons to consider how site-specific contextual factors interact with the variation in the intensity and fidelity of implementation to affect teamwork and patient outcomes.

**Discussion:**

The RESET study provides mentorship and resources to assist hospitals as they implement complementary and mutually reinforcing components to redesign the clinical microsystems caring for medical patients. Our findings will be of interest and directly applicable to all hospitals providing care to patients with medical conditions.

**Trial registration:**

NCT03745677. Retrospectively registered on November 19, 2018.

**Electronic supplementary material:**

The online version of this article (10.1186/s12913-019-4116-z) contains supplementary material, which is available to authorized users.

## Contributions to the literature

- Most adults requiring hospitalization are admitted for medical conditions, yet the optimal model of care for these patients is yet to be established.
- This study uses a clinical microsystems framework and a set of complementary, mutually reinforcing interventions to redesign systems of care. Prior studies have generally evaluated single interventions.
- This study will evaluate the effect of the interventions on teamwork climate and adverse events. Few studies evaluating similar interventions have assessed patient safety measures.
- This study also fills a gap in the literature by assessing how site-specific contextual factors influence adaptation of the interventions and the success of implementation.

## Background

Despite major efforts over the past two decades, the evidence suggests that we are still a long way from consistently delivering high quality care to hospitalized patients [1, 2]. Most adults requiring hospitalization are admitted for medical conditions [3], yet the optimal model of care for these patients is yet to be established [4]. Teams caring for medical patients are large, with membership that continually evolves and is seldom in the same place at the same time [5]. Physicians are often spread across multiple units and floors giving them little opportunity to develop relationships with nurses and other professionals who work on designated units [6]. Nurse and physician leaders commonly operate in silos, limiting their ability to address challenges collaboratively [7]. Patients and family members are generally poorly informed and lack opportunities to engage in decision making about their care [8]. As a result, medical services lack the structure and professionals lack the shared accountability necessary to optimally coordinate care on a daily basis and improve performance over time [9].

A growing body of research has tested interventions to redesign aspects of the care delivery system for hospitalized medical patients. These interventions include:

- *Localization of physicians.* Studies have shown that localizing physicians to specific units increases the frequency with which nurses and physicians discuss their patients’ plans of care and reduces the number of pages received by physicians, presumably due to greater face-to-face communication [10, 11].
- *Unit nurse-physician co-leadership.* Unit nurse-physician co-leadership is a collaborative model in which a nurse leader and physician leader share responsibility for quality on their unit [7]. Though not rigorously evaluated, the model has been associated with reductions in Central Line-Associated Blood Stream Infections, Catheter Associated Urinary Tract Infections, and pressure ulcers [12].
- *Interprofessional rounds (a.k.a., multidisciplinary rounds and interdisciplinary rounds).* Systematic reviews have evaluated the impact of interprofessional rounds in medical settings and found evidence to support improvements in staff satisfaction, but an inconsistent effect on length of stay [13, 14]. Though the evidence suggests improvements in patient safety, few studies have evaluated the effect of interprofessional rounds on adverse events. An important development is the growing use of interprofessional rounds at the bedside (a.k.a., patient and family centered rounds) to better inform and engage patients. The model is common in pediatric settings [15], but few studies have evaluated the impact in adult settings [16, 17].
- *Performance dashboards.* Individuals may struggle to understand how their work is connected to the organization’s overall performance. To ensure meaning and accountability at all levels, some leaders have developed group and unit level performance dashboards [18, 19].
- *Patient engagement strategies*. Patient engagement is associated with fewer adverse events and hospital readmissions [20, 21]. Unit-based patient engagement strategies include use of white boards in patient rooms to establish goals and communicate the plan of care, patient experience rounds by local leaders, and conducting nurse shift-change report as well as interprofessional rounds at the bedside. Despite these practices having strong face validity, research shows they are not used consistently [22].

Importantly, the overwhelming majority of prior research studies have evaluated the effect of a single intervention (e.g., physician localization without unit nurse-physician co-leadership or interprofessional rounds) [10, 13, 23]. We believe these interventions are better conceptualized as complementary and mutually reinforcing components of a redesigned *clinical microsystem* and should be implemented and evaluated as such. A *clinical microsystem* is defined as the small group of people who work together in a defined setting on a regular basis to provide care [24, 25]. The benefit of using complementary interventions to redesign the microsystems which care for medical patients is not known. Furthermore, the influence of contextual factors has not been determined, nor have we identified strategies associated with successful adaptation and implementation.

In an effort to establish and disseminate the optimal model of care to improve outcomes for hospitalized patients, we received Agency for Healthcare Research and Quality (AHRQ) funding for the **RE**designing **S**yst**E**ms to Improve **T**eamwork and Quality for Hospitalized Patients (RESET) study. Our specific objectives for this study is to implement a set of evidence-based complementary interventions across a range of clinical microsystems, identify factors and strategies associated with successful implementation, and evaluate the impact on quality. RESET uses a parallel group study design and will use two group pretest-posttest analyses for primary outcomes.

The specific aims for the RESET study are to:

1. Conduct a multi-site mentored implementation study in which each site adapts and implements complementary interventions to improve care for medical patients.
2. Evaluate the effect of the intervention set on teamwork climate and patient outcomes related to safety, patient experience, and efficiency of care for hospitalized medical patients.
3. Assess how site-specific contextual factors interact with the variation in the intensity and fidelity of implementation to affect teamwork and patient outcomes.

## Methods/design

### Clinical microsystem framework

Research has identified 5 overarching characteristics associated with successful microsystems: local leadership, focus on the needs of staff, emphasis on the needs of patients, attention to performance, and a rich information environment. Medical services provide an ideal opportunity to study the impact of redesigning clinical microsystems because challenges exist in each of these 5 areas (Table 1). Furthermore, improvements in these areas may impact care across a range of conditions, not just for a particular diagnosis.

**Table 1:** Challenges on Medical Services by Microsystem Domain

Domains	Challenges
Local Leadership	• Nursing and physician leaders often operate in silos. • Physician leadership at the unit level may not exist. • Formal training of unit leaders is often lacking.
Focus on Needs of Staff	• Dispersion of physicians limits their connection to any particular unit. • Team members inconsistently given orientation to units/services. • Team member roles and expectations not defined.
Emphasis on Needs of Patients	• Patients have poor comprehension of plan of care. • Limited opportunities exist for patients and families to partner in care.
Attention to Performance	• Performance data often unavailable at the unit level. • Limited data to prompt changes during patients’ hospitalizations.
Rich Information Environment	• Few opportunities for team members to share information and collaborate on better decisions. • Technology not leveraged to identify opportunities to improve care.

### The Advanced and Integrated MicroSystems (AIMS) interventions

The RESET project uses the Advanced and Integrated MicroSystems (AIMS) interventions, complementary and mutually reinforcing components of a redesigned clinical microsystem. The AIMS interventions address the challenges previously described and consist of 1) Unit-based Physician Teams, 2) Unit Nurse-Physician Co-leadership, 3) Enhanced Interprofessional Rounds, 4) Unit-level Performance Reports, and 5) Patient Engagement Activities. Our research team developed the AIMs interventions from available evidence, a detailed needs assessment, and our research team’s past experience implementing similar interventions [4, 26–28]. Each intervention is supported by specific processes and tools (Table 2). Importantly, many hospitals have implemented some of these interventions, but implementation is often incomplete and few have implemented all components [29].

**Table 2:** Advanced and Integrated MicroSystems (AIMS) Interventions, Supporting Processes and Tools

Components	Descriptions	Supporting Processes and Tools
Unit-based Physician Teams	Localization of physicians to a minimal number of units on which they provide patient care	• Projecting expected patient volume • Engaging stakeholders to redesign admission processes • Monitoring progress and making adjustments
Unit Nurse-Physician Co-leadership	Collaborative model in which a nurse leader and physician leader are jointly responsible for quality improvement on their unit	• Co-leader selection and training • Co-leader job descriptions and activities • Establishing unit norms and values • Co-leader integration into mesosystem activities
Enhanced Interprofessional Rounds	Interprofessional rounds, redesigned with input from frontline professionals to optimize collaboration and patient engagement	• Redesign work groups determine timing, format, duration, and location • Discussions facilitated by unit co-leaders • Roles / expectations of attendees defined • Structured tools to support closed-loop communication
Unit-level Performance Reports	Performance reports designed to give unit leaders and frontline professionals relevant, interpretable, actionable data	• Monthly unit-level reports aligned with organizational priorities • Daily reports to identify opportunities to improve care • Just-in-time reports to identify opportunities to improve care • Teamwork Climate survey reports
Patient Engagement Activities	Methods to continually inform and engage patients and families as partners in care	• Use of whiteboards to define goals and the daily care plan • Patient experience rounds by unit co-leaders • Conducting Interprofessional Rounds and nurse shift reports at bedside

### Study sites

In collaboration with the Society of Hospital Medicine (SHM) and the American Nurses Association (ANA), we issued a national call for applications for the RESET project. We received 14 applications from hospitals throughout the U.S, each of which was independently assessed by two members of research team for need (i.e., similar interventions had not already been implemented), commitment, and potential for success. Four hospital sites were selected, with two hospitals in the Southeast U.S., one in the Midwest, and one in the West. All hospitals are nonprofit and have between 200 and 350 beds. Two are non-teaching hospitals and two are teaching hospitals, though neither is a major affiliate of a medical school.

### Mentored implementation, site leaders, and site project teams

This study uses SHM’s mentored implementation model, which involves coaching provided by external professionals who are practicing experts in the area of focus [30]. Mentors develop an understanding of the hospital and culture, provide guidance in developing and implementing operational work plans, share their own experiences and strategies for overcoming barriers, encourage the teams to adhere to the timelines, and teach techniques for facilitating effective practice change. RESET involves two mentorship teams, each consisting of a physician and nurse with experience in leading the redesign of clinical microsystems. Mentors received 6 h of SHM Mentor University training for their role, which occurred during an in-person meeting at SHM headquarters and included an overview of the study aims, scope, and methods, fundamentals of mentoring, and mentor expectations [30].

Each study site has assembled a local leadership team, including a physician leader, a nurse leader, and a research nurse. Site physician and nurse leaders dedicate sufficient time for the study with support from their hospital. The research nurse receives funding from the grant to support effort for data collection and local project management activities. Mentors coach sites during monthly calls with site leadership teams. The research team hosts monthly calls with all mentors, during which each mentor team provides updates on sites’ progress. Each site also received guidance from their mentor team through an initial two-day site visit to assess relationships with key stakeholders, site infrastructure, and readiness for change. Mentor teams provided a written report with observations from site visits and recommendation to site leaders.

We also convene all site leaders in a webinar thrice in year one and twice annually thereafter. During the webinars, sites share their progress, adaptations, and lessons to date. Site leaders also receive feedback from one another and from the research team.

### Implementation framework

**Fig. 1:**
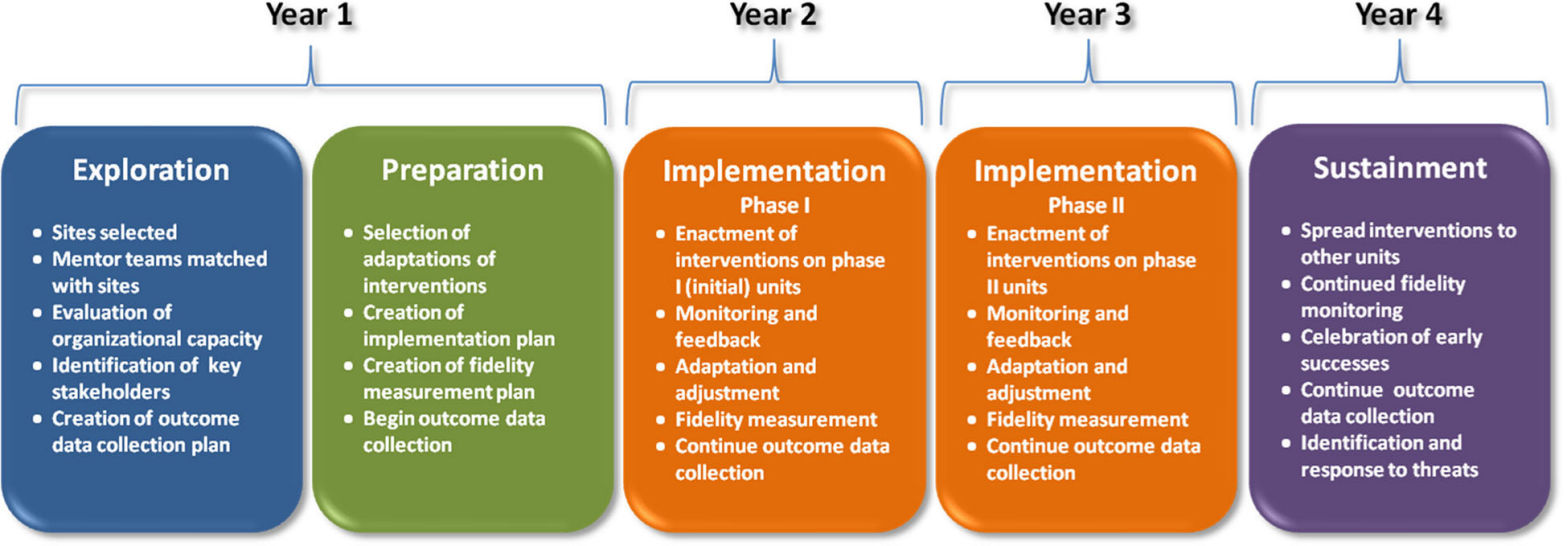
Overview of Implementation Approach using EPIS Framework

The Exploration, Preparation, Implementation, and Sustainment (EPIS) model guides both our implementation approach and our evaluation (Figure 1) [31]. The EPIS model was developed by Aarons and colleagues and has been used extensively to identify variables which play important roles in achieving effective implementation of evidence-based practices [32, 33]. Importantly, the EPIS model recognizes that different variables play crucial roles at different points in the implementation process.

#### Exploration and preparation

Exploration began with solicitation of potential sites, review of site applications, and selection of final study sites. Mentors were matched with sites and began coaching to assist site leaders in engaging stakeholders and selecting specific adaptations of interventions to meet their needs. We prepared and distributed a 52 page RESET Implementation Guide to site leaders, which includes detailed descriptions of each AIMS intervention, recommended strategies for successful implementation, milestones, and tools (e.g., an example project charter, work plan and communication plan templates, roles and expectations of unit co-leaders, and example structured communication tools to be used in Enhanced Interprofessional Rounds).

During Preparation, mentors assisted site leaders as they conducted a formal evaluation of organizational capacity for planned interventions and created an implementation plan. In collaboration with site leaders, we administered the Organizational Readiness for Implementing Change (ORIC) survey to all professionals on the study units [34]. ORIC is a 12 item instrument based on Weiner’s theory of organizational readiness for change and measures two core constructs: change commitment and change efficacy [35] ORIC is ideal for our study because it is brief, reliable, and items are understood by frontline hospital based professionals [34]. The ORIC results were shared with site leaders and mentors led discussions reviewing the results during monthly calls.

**Fig. 2:**
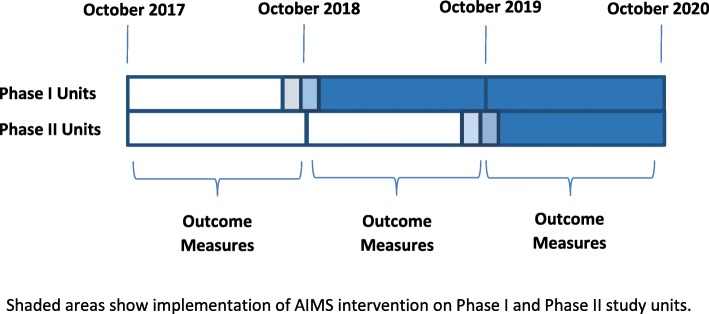
Overview of RESET Study Design and Data Collection

In preparing the implementation plan, site leaders selected 1 unit ideally suited for initial implementation of interventions (Phase I Implementation) and 1–2 units for later implementation of interventions (Phase II Implementation). Implementation of the AIMS interventions on the Phase I units was planned to occur in or around October 2018 and implementation on the Phase II units is to occur in or around October 2019. Outcome data are collected for both Phase I and Phase II units before and after implementation of the AIMS interventions on the Phase I units (See Figure 2).

Site leaders also assembled RESET project teams to meet every other week, with recommended membership consisting of the site project leaders, unit co-leaders, hospital quality improvement leaders, frontline professionals, and patient/family member representatives. Site leaders were advised to include other key stakeholders as needed, including professionals from bed assignment, emergency medicine, information technology, professional development, and patient experience.

#### Implementation

During Implementation Phase I, the AIMS interventions are implemented on the initial, phase I Implementation units. The research nurse conducts fidelity measurement for phase I implementation units and continues outcome data collection for all study units. During Implementation Phase II, interventions will be implemented on additional, phase II implementation units, leveraging lessons learned during phase I. Fidelity measurement and outcome data collection will continue for all study units. Our use of a phased approach during implementation is consistent with prior studies using similar interventions, [26, 28, 36] allows lessons learned in early implementation to be incorporated into later implementation efforts, and provides for a rigorous evaluation using both historic and concurrent controls (i.e., difference-in-differences analytic strategy).

#### Sustainment

During Sustainment, site leaders will continue to monitor fidelity measures and make needed adjustments to Implementation Phase I and II units. Site leaders will continue to spread interventions to other units, as appropriate, though no formal evaluation of fidelity will occur beyond Phase I and Phase II units. Mentors will assist site leaders in identifying and responding to potential threats, including bed capacity constraints, development of new clinical services, staffing shortages, and other competing priorities.

### Data collection

Each site has designated a research nurse who receives grant funding to support effort to conduct observations, help administer surveys to professionals, conduct medical record abstractions, and assemble data from administrative databases. The research nurse provides data to the central coordinating center (Northwestern University) via the Research Electronic Data Capture (REDCap) platform, a secure, HIPAA compliant web-based application designed to support data capture for research studies [37].

### Fidelity measures

Fidelity data are collected by the research nurse during brief interviews of physicians, brief surveys of hospital leaders, and direct observations (See Additional file 1: Table S1). The exact timing of observations is not disclosed to healthcare professionals (i.e., interviews and observations will be unannounced) to reduce risk for bias. Monthly reports of fidelity measure performance are provided by the research team to site leaders and their mentors. Monitoring of fidelity measures and ongoing mentorship allows site leaders to make adjustments to optimize implementation.

### Outcome measures

#### Teamwork climate (primary outcome)

We will assess teamwork climate using the Safety Attitudes Questionnaire (SAQ) developed by Sexton et al. [38] The SAQ teamwork climate domain includes 14 questions, generates a score from 0 to 100, and has been sensitive to change in prior studies assessing microsystem change [36, 39]. Similar to prior studies, we also ask respondents to rate the quality of collaboration experienced with each professional type [6, 40]. We administer the survey annually (years 1 through 4) to all nurses, nurse assistants, physicians, pharmacists, social workers, and case managers on study units. Names and email addresses of professionals on study units are obtained and the survey administered by the central coordinating center using REDCap. Site leaders promote completion of the survey and non-responders receive up to 5 reminder emails to optimize response rates.

#### Adverse events (primary outcome)

We use the Medicare Patient Safety Monitoring System (MPSMS) methodology to detect adverse events. MPSMS is a medical record-based national patient safety surveillance system that provides rates for specific inpatient adverse event measures [41, 42]. MPSMS data have been used in Agency for Healthcare Research and Quality National Health Care Quality and Disparities Reports and currently serve as the major national-level patient safety data source for the U.S. Department of Health and Human Services [1]. MPSPS has been shown be reliable and valid in detecting adverse events among hospitalized patients [41, 43]. We collect data for 9 types of adverse events which commonly occur among hospitalized general medical patients, including adverse drug events, hospital acquired infections, pressure ulcers, and falls. Data will be reported as the number and percentage of patients experiencing one or more adverse event and the number of adverse events per 1000 discharges.

#### Patient experience (secondary outcome)

We use Hospital Consumer Assessment of Healthcare Providers and Systems (HCAHPS) global ratings of hospital care [44]. Data will be reported as the number and percentage of patients giving the most favorable rating (“top box”) to patient satisfaction questions. Each year, the research nurse will obtain data for patients admitted to study units, excluding those transferred from other hospitals and those initially admitted to other units. Research nurses will de-identify data prior to importing it into REDcap.

#### Efficiency measures (secondary outcomes)

We assess efficiency of care using hospital length of stay (LOS) and 30-day readmissions. LOS will be reported as median (IQR) as data are typically skewed in distribution. As for patient experience data, the research nurse will obtain data on a yearly basis for patients admitted to study units, excluding those transferred from other hospitals and those initially admitted to other units. Research nurses will de-identify data prior to importing it into REDcap. This data will include information on patient age, sex, race, payer, admission source, primary diagnosis, secondary diagnoses, type of admission (i.e., observation vs. inpatient), and discharge destination (i.e., home vs. other).

### Statistical analyses

Research team members at Northwestern University (KJO and JL) will have access to quantitative data and conduct analyses. We will conduct several analyses for each of our two primary outcomes: teamwork climate and adverse events.

#### Teamwork climate

First, we will compare baseline teamwork climate scores to post-implementation scores for Phase I Implementation Units. We will use two-sample t tests (for all professionals) and paired t tests (for professionals responding to both surveys). Data is expected to be normally distributed based on prior studies [36, 39]. Second, we will compare baseline teamwork climate scores to post-implementation scores for Phase II Implementation Units using two-sample t tests (for all professionals) and paired t tests (for professionals responding to both surveys). Third, we will conduct a two group pretest-posttest analysis using multiple linear regression with teamwork score as the outcome variable. The model will compare the change in teamwork climate from baseline to post-implementation in Phase I Implementation units to the change in Phase II Implementation units across 2 baseline periods. The model will use an interaction term defined as unit type (Phase I vs. Phase II) multiplied by the intervention period (year 1 vs. year 2) to examine the change in teamwork climate (i.e., difference-in-differences analysis). We will use standard errors robust to the clustering of professionals within each hospital.

#### Adverse events

We will conduct two group pretest-posttest analyses using 2 multiple regression models. The first model will use logistic regression and the occurrence of one or more adverse events as the outcome variable (0 if patient experiences no adverse events, 1 if the patient experiences ≥1). The model will compare the change in the percentage of patients experiencing one or more adverse events from baseline to post-implementation in Phase I Implementation units to the change in Phase II Implementation units across 2 baseline periods. The model will use an interaction term defined as unit type (Phase I vs. Phase II) multiplied by the intervention period (year 1 vs. year 2) to examine the change in the outcome. (i.e., difference-in-differences analysis). We will use standard errors robust to the clustering of patients within each hospital. The second model will use Poisson regression and the number of adverse events per 1000 discharges as the outcome variable. This method accounts for episodes in which a patient may experience more than one adverse event. This model will compare the change in the number of adverse events per 1000 discharges from baseline to post-implementation in Phase I Implementation units to the change in Phase II Implementation units across 2 baseline periods. The model will use an interaction term similar to that described above (i.e., difference-in-differences analysis) and include standard errors robust to the clustering of patients within each hospital. Covariates for both models will include patient age, sex, race, payer, admission source, primary diagnosis, Elixhauser index [45], type of admission (i.e., observation vs. inpatient), and discharge destination (i.e., home vs. other).

### Power and sample size

#### Teamwork climate

Prior research using similar interventions has shown that teamwork climate increases for all professionals, but the improvement is greatest for nurses [36, 39, 40]. Therefore, we estimated a target sample size of nurses using results from prior studies. A sample size of 68 achieves 90% power to detect a mean of paired differences of 4.0 with an estimated standard deviation of differences of 10.0 and with a significance level of 0.05 using a two-sided paired t-test [46]. This sample size reflects completed paired surveys for 17 nurses at each site and is extremely feasible based on prior studies [36, 39, 40].

#### Adverse events

We used data from the AHRQ National Scorecard on Rates of Hospital Acquired Conditions 2010 to 2015, which also uses the MPSMS methodology, to estimate current rates for the adverse events included in our study [47]. The national rate for adverse events included in our study is 89.3 per 1000 discharges. Based on prior studies, we conservatively expect a 33% reduction in the rate of adverse events as a result of our interventions [36, 48]. Importantly, adverse events have recently declined by approximately 4% each year [47]. Accounting for this trend, we estimate a need to enroll a target sample size of 1936 patients each year (482 per site per year) to have 90% power to detect a significant improvement in adverse events using a Poisson regression accounting for clustering of hospitals (r = 0.3).

### Qualitative data collection

We use a multi-method approach to collect and triangulate data from numerous sources at each of the study sites. Site visits form the basis of the data collection and provide the opportunity to conduct ethnographic observations, semi-structured interviews, and focus groups.

#### Site visits

An interdisciplinary site visit team consisting of a qualitative researcher (JKJ), a physician-researcher (KJO), and a nurse-researcher (MM) conducts the site visits. Site visits are conducted at each site in years 1, 3, and 5. Data are collected through observations, interviews, and focus groups.

#### Observations

We conduct a series of ethnographic observations during the site visits to help us understand each hospital’s local context and how unit-specific contextual factors affect the adaptation and implementation of the intervention components [49, 50] (e.g., observe physician and nurse work activities, interprofessional rounds) (see Additional file 2). In addition, we tour the medical units to observe physical spaces and have conversations with hospital staff in their own environment. The site team assumes the role of “peripheral-member-researcher” which brings an insider’s perspective to the observations to allow accurate appraisal of activities [51].

#### Interviews

We conduct semi-structured interviews with key stakeholders at each site [52, 53] (see Additional file 3). Participants include the site leadership team (physician leader, nurse leader, and research nurse), Chief Medical Officer, and Chief Nurse Officer. Interview data is an important source of the information about how individuals think about quality, local culture, and the effectiveness of the interventions. Interview questions focus on assessing the local adaptation and implementation of each component, as well as the individual’s perceptions of the utility of each component.

#### Focus groups

We conduct focus groups in each hospital using a set of semi-structured questions to guide discussion. Focus group participants include the site leadership team, hospitalists, nurses, and the hospital professionals involved in implementation at the microsystem level (e.g., unit co-leaders). The data from the focus groups provides important information about issues related to each intervention and how site team members interact with one another.

#### Artifacts

Artifacts are “the implements, notes, or materials” used during the adaptation and implementation of the interventions (e.g., hospital application, notes from mentor calls, structured communication tools, unit performance reports) [54]. Analysis of these artifacts will provide additional information about site-specific contextual factors [55].

#### Qualitative data analysis

Each researcher takes detailed notes of observations and discussions during interviews and focus groups. Notes will be analyzed using a computer-assisted qualitative data analysis software (MAXQDA). During analysis, categorical themes will be identified and applied. We will conduct a hybrid form of analysis which will combine inductive and deductive logics [56, 57]. The analytic strategy is informed by the task at hand (evaluation of the adaptation and implementation of the interventions), as well as the desire to allow unanticipated themes to emerge from the data and to allow participants’ understandings to come to the fore [58]. Deductively, we will begin by imposing a priori categories that serve the needs of the evaluation. While the first dimension is chronologically ordered (Exploration, Preparation, Implementation, Sustainment), the second dimension adapts the conceptual framework for analyzing textual data proposed by Corbin and Strauss [59]. We will use cross-case comparisons to consider how site-specific contextual factors interact with the variation in the intensity and fidelity of implementation to affect teamwork and patient outcomes [60]. Generalizability will be increased by providing details of the changes and contextual factors of each hospital.

### Timeline

**Fig. 3:**
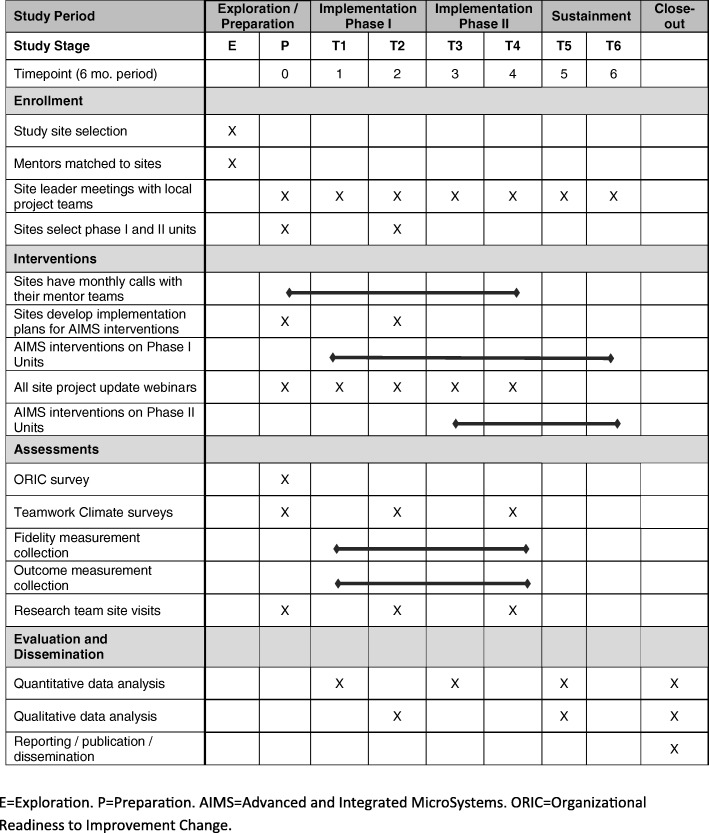
RESET Study Timeline (SPIRIT diagram of trial stages of enrollment, intervention, assessment, and evaluation)

A high level timeline for the RESET study is provided in Figure 3. Exploration and Preparation occurred in year 1. Sites began to implement the AIMS interventions on Phase I units in year 2 (Implementation Phase I). Sites implement the AIMS interventions on Phase II units in year 3 (Implementation Phase II). Sustainment occurs in year 4 and program analysis occurs in year 5.

## Discussion

A number of challenges impede our ability to consistently provide high quality care to patients with medical problems. The RESET study uses a clinical microsystems framework and a set of complementary, mutually reinforcing interventions to redesign systems of care for hospitalized medical patients. Quantitative data will allow us to evaluate the effect the interventions on teamwork climate and patient outcomes, while qualitative data will allow us to assess how site-specific contextual factors influence adaptation of the interventions and influence the success of implementation.

### Strengths

The RESET study has several notable strengths. First, the majority of prior studies have evaluated the effect of single interventions to redesign aspects of care delivery for patients hospitalized with medical conditions. RESET will assess the effect of a set of complementary and mutually reinforcing interventions (i.e., the AIMS interventions). Second, RESET uses an established mentored implementation approach and interprofessional site and mentor teams. Third, our phased implementation allows lessons learned in early implementation to be incorporated into later implementation efforts, and provides for a rigorous evaluation using both historic and concurrent controls (i.e., difference-in-differences analytic strategy). Most prior studies have used a before-and-after study design which does not account for baseline trends in outcomes assessed. Fourth, we provide resources for collection of fidelity and outcome measures. Providing fidelity measures to sites on a regular basis allows them to identify areas for improvement and make adaptations. Finally, our qualitative assessment will allow us to describe how sites have adapted the interventions and contextual factors which influenced the success of implementation.

### Challenges

We anticipated several challenges with the RESET study. Even with the rigorous support provided, sites have found the redesign of hospital systems to be difficult. The biggest challenges thus far relate to unit-based physician teams, unit nurse-physician co-leadership, and enhanced interprofessional rounds. With regard to unit-based physician teams, sites have had to make fundamental changes to their processes for assigning newly admitted patients to physicians. Sites are now designating specific physicians to care for patients on specific units and newly admitted patients are assigned to a physician based on their unit location.

The implementation of unit nurse-ohysician co-leadership has been challenging because sites have struggled to provide protected time for unit physician leaders to serve in this role. Additionally, some sites have felt that they have few talented and/or interested physician leaders to serve as a unit physician leader. Mentors have coached teams in advocating for this role and selection and training of potential physician leaders.

Of note, all sites have decided to implement enhanced interprofessional rounds at the bedside. Site teams have had to address key issues when planning the implementation of enhanced interprofessional rounds, including the determination of which professionals should be present, the optimal duration, and whether interprofessional rounds should be combined with the physician’s planned encounter for the day. Regarding the latter, most sites have planned to have physicians visit patients before interprofessional rounds to discuss more detailed medical issues and perform physical examinations.

### Dissemination and impact

Findings from this study will be directly applicable to all hospitals caring for patients with general medical conditions. Importantly, the study was informed by, and leverages the expertise and influence of a diverse group of stakeholders, including SHM, the ANA, and the Institute for Patient- and Family-Centered Care. These stakeholder groups continue to provide critical input and will be instrumental in disseminating our findings.

## Additional files

Additional file 1: **Table S1.** Measures to Assess Fidelity of Implementation. **Table S2.** Safety, Patient Experience, and Efficiency Outcome Measures (DOCX 14 kb)

Additional file 2: Redesigning Systems to Improve Teamwork and Quality for Hospitalized Patients (RESET) – Site Visit. Observation protocol (DOCX 13 kb)

Additional file 3: Redesigning Systems to Improve Teamwork and Quality for Hospitalized Patients (RESET) – Site Visit. Interview guide for semi-structured interviews with leaders and guide for focus group discussions with front line staff (DOCX 38 kb)

## Abbreviations

AIMS: Advanced and Integrated MicroSystems
ANA: American Nurses Association
EPIS: Exploration, Preparation, Implementation, Sustainment
HCAHPS: Hospital Consumer Assessment of Healthcare Providers and Systems
MPSMS: Medicare Patient Safety Monitoring System
ORIC: Organizational Readiness for Implementing Change
RESET: Redesigning Systems to Improve Teamwork and Quality for Hospitalized Patients
SAQ: Safety Attitudes Questionnaire
SHM: Society of Hospital Medicine
