# Supplementary material for: Redesigning systems to improve teamwork and quality for hospitalized patients (RESET): study protocol evaluating the effect of mentored implementation to redesign clinical microsystems
Source: BMC Health Serv Res. 2019 May 8;19:293. doi: 10.1186/s12913-019-4116-z (PMC6505207; doi:10.1186/s12913-019-4116-z)
Supplement: Supplementary file 1 — Table S1. Measures to Assess Fidelity of Implementation. Table S2. Safety, Patient Experience, and Efficiency Outcome Measures (DOCX 14 kb) [file 12913_2019_4116_MOESM1_ESM.docx]

**APPENDIX**

**Appendix Table 1.** Measures to Assess Fidelity of Implementation

| **Intervention Component** | | **Description** | **Data Collection (Frequency)** |
| --- | --- | --- | --- |
| **Unit Based Physician Teams** | | | |
|  | Physician units | Number of units each physician cares for patients | Unannounced interviews (10 / mo.) |
|  | Percent localized | Percentage of physicians’ patients on designated unit | Unannounced interviews (10 / mo.) |
| **Nurse-Physician Co-leadership** | | | |
|  | Co-leadership model | Presence of nurse-physician co-leadership model for each unit | Brief survey of hospital leaders (monthly) |
|  | Medical Director effort | Percent of effort unit medical director has for role | Brief survey of hospital leaders (monthly) |
| **Enhanced Interprofessional Rounds (IPR)** | | | |
|  | Frequency of IPR by unit | Number of times IPR is completed per week | Unannounced direct observations (10 / mo.) |
|  | Presence of physicians | Presence of physicians at IPR (present for no, some, or all patients) | Unannounced direct observations (10 / mo.) |
|  | Presence of nurses | Presence of nurses at IPR (present for no, some, or all patients) | Unannounced direct observations (10 / mo.) |
|  | Presence of pharmacists | Presence of pharmacist at IPR (present for no, some, or all patients) | Unannounced direct observations (10 / mo.) |
|  | Location of IPR | Location of IPR (bedside, hallway, conference room, other) | Unannounced direct observations (10 / mo.) |
| **Unit-level Performance Reports** | | | |
|  | Unit level dashboard | Availability of performance dashboard at unit level | Review of actual report (monthly) |
|  | Near real time reports | Presence of ≥1 near real time report (e.g., sepsis alert) | Review of actual report (monthly) |
| **Patient Engagement Activities** | | | |
|  | Provider names on whiteboard | Correct names of nurse and primary physician on whiteboard | Unannounced direct observations (10 / mo.) |
|  | Goals on whiteboard | Presence of ≥ 1 patient goal on whiteboard | Unannounced direct observations (10 / mo.) |
|  | Patient experience rounds | Number of times unit co-leaders perform patient experience rounds | Brief survey of hospital leaders (monthly) |
|  | Nursing bedside reports | Nurse shift reports conducted at bedside (never, sometimes, always) | Brief survey of hospital leaders (monthly) |

IPR = Interprofessional Rounds

**Appendix Table 2.** Safety, Patient Experience, and Efficiency Outcome Measures

| **Outcome Category** | | | | **Data Source** |
| --- | --- | --- | --- | --- |
| **Safety** | | | | |
|  | Adverse Drug Events (ADE) associated w/ | | |  |
|  |  | | Hypoglycemic agent | Medical record abstraction using MPSMS |
|  |  | | IV Heparin | Medical record abstraction using MPSMS |
|  |  | | Low Molecular Weight Heparin or Factor Xa Inhibitor | Medical record abstraction using MPSMS |
|  |  | | Warfarin | Medical record abstraction using MPSMS |
|  | Hospital Acquired Infection | | |  |
|  |  | | Catheter-Associated Urinary Tract Infection | Medical record abstraction using MPSMS |
|  |  | | Central Line-Associated Blood Stream Infection | Medical record abstraction using MPSMS |
|  |  | | Hospital Acquired Antibiotic-Associated *C. difficile* | Medical record abstraction using MPSMS |
|  | Hospital-Acquired Pressure Ulcers | | | Medical record abstraction using MPSMS |
|  | In-Hospital Patient Falls | | | Medical record abstraction using MPSMS |
| **Patient Experience** | | | | |
|  | | Likelihood to recommend | | Administrative data using HCAHPS |
|  | | Overall hospital rating | | Administrative data using HCAHPS |
| **Efficiency** | | | | |
|  | | Length of stay | | Administrative data |
|  | | 30 day readmission | | Administrative data |

MPSMS = Medicare Patient Safety Monitoring System

HCAHPS = Hospital Consumer Assessment of Healthcare Providers and Systems
