# Supplementary material for: Redesigning systems to improve teamwork and quality for hospitalized patients (RESET): study protocol evaluating the effect of mentored implementation to redesign clinical microsystems
Source: BMC Health Serv Res. 2019 May 8;19:293. doi: 10.1186/s12913-019-4116-z (PMC6505207; doi:10.1186/s12913-019-4116-z)
Supplement: Supplementary file 2 — Redesigning Systems to Improve Teamwork and Quality for Hospitalized Patients (RESET) – Site Visit. Observation protocol (DOCX 13 kb) [file 12913_2019_4116_MOESM2_ESM.docx]

**Redesigning Systems to Improve Teamwork and Quality for Hospitalized Patients**

**(RESET) – Site Visit**

**Observation Protocol**

**Time:**

**Date: Location: Researcher:**

| **Descriptive Notes** | **Reflective Notes** |
| --- | --- |
| Description of the physical settings  Reconstruction of dialogue  Accounts of particular events  Accounts of particular activities | Researcher thoughts/impressions about the  descriptive notes |
