# Supplementary material for: Redesigning systems to improve teamwork and quality for hospitalized patients (RESET): study protocol evaluating the effect of mentored implementation to redesign clinical microsystems
Source: BMC Health Serv Res. 2019 May 8;19:293. doi: 10.1186/s12913-019-4116-z (PMC6505207; doi:10.1186/s12913-019-4116-z)
Supplement: Supplementary file 3 — Redesigning Systems to Improve Teamwork and Quality for Hospitalized Patients (RESET) – Site Visit. Interview guide for semi-structured interviews with leaders and guide for focus group discussions with front line staff (DOCX 38 kb) [file 12913_2019_4116_MOESM3_ESM.docx]

**Redesigning Systems to Improve Teamwork and Quality for Hospitalized Patients (RESET) - Site Visit**

**Interview Guide for Semi-Structured Interview with Leaders**

**Conducted by Research Team**

**Participants: Senior Leaders and Site Leaders**

**Introduction:**

Thank you for agreeing to talk with us today. We are conducting interviews with several people to learn about your hospital’s experience in participating in the RESET project. We are particularly interested in identifying any facilitators or potential barriers to implementation.

After our site visit we will summarize the interviews and provide feedback to the hospital. Your comments will be anonymous, so your specific comments will not be identifiable.

We would like to record the interview because that makes it easier for us to focus on the conversation instead of taking notes. Is it okay with you if we record our conversation?

Do you have any questions for us before we start?

1. Let’s start by talking about quality of care for hospitalized medical patients. What can you tell us about the quality of care for general medical patients in your hospital?

2. Culture can be defined as the product of individual and group values, attitudes, perceptions, competencies, and patterns of behavior. What is the culture like on [study unit]? How does the culture differ across units?

3. How do you think the RESET project will help improve quality for medical patients at your hospital? What aspects of the project will help to improve quality? Please provide an example.

4. How do you think the RESET project will help improve culture?

5. The RESET project includes 5 intervention components: 1) Unit-based Physician Teams, 2) Unit Nurse-Physician Co-leadership, 3) Enhanced Interprofessional Rounds, 4) Unit-level Performance Reports, 5) Patient Engagement Activities. What do you think will be the main benefits to implementing these interventions?

6. What do you think will be the most important challenges to implementing the intervention components?

7. What steps are being taken to help address those challenges? What are some strategies that your hospital is considering putting in place to make it easier for the interventions to be implemented?

8. The RESET project includes several approaches that we think will help implementation, including mentor teams, education and training, measures of fidelity, measures of teamwork and outcomes.

What do you think will be most helpful and why?

Do you think there are things that will not be especially helpful?

What other approaches should we consider to help improve the success of this project?

9. What have you done to disseminate information about the RESET project?

10. What would you like to share with other RESET sites?

Prompt: What do you think your hospital is doing well, what makes you proud?

Tell us what is keeping you up at night?

**Conclusion:**

Is there anything else that you would like to tell us about, or make sure that we learn about while we are here today?

Thanks for agreeing to take time to meet with us today . . . .

**Redesigning Systems to Improve Teamwork and Quality for Hospitalized Patients (RESET) - Site Visit**

**Guide for Focus Group Discussions with front line staff**

**Participants: Front line nurses, hospitalists, pharmacists, social workers, case managers, and therapists**

**Introduction:**

Thank you for agreeing to talk with us today. We are conducting focus groups with hospital professionals to learn about your hospital’s experience in participating in the RESET project. We are particularly interested in identifying any facilitators or potential barriers to implementation.

After our site visit we will summarize the interviews and provide feedback to the hospital. Your comments will be anonymous, so your specific comments will not be identifiable.

We would like to record the interview because that makes it easier for us to focus on the conversation instead of taking notes. Is it okay with you if we record our conversation?

Do you have any questions for us before we start?

_____________

1. Let’s start by talking about quality of care for hospitalized medical patients. What can you tell us about the quality of care for general medical patients on your unit?

2. Culture can be defined as the product of individual and group values, attitudes, perceptions, competencies, and patterns of behavior. What is the culture like on [study unit]? How does culture differ across the units?

3. How do you think the RESET project will help improve quality for medical patients on your unit(s)?

4. How do you think the RESET project will help improve culture on your unit(s)?

5. The RESET project includes 5 intervention components: 1) Unit-based Physician Teams, 2) Unit Nurse-Physician Co-leadership, 3) Enhanced Interprofessional Rounds, 4) Unit-level Performance Reports, 5) Patient Engagement Activities. What do you think will be the main benefits to implementing these interventions?

6. What do you think will be the most important challenges to implementing the intervention components?

7. What steps are being (should be) taken to help address those challenges? What are some strategies that your hospital is considering putting in place to make it easier for the interventions to be implemented?

8. In what ways do you learn about RESET project?

Prompts: What are some of the ways the project leaders team has informed you of next steps in the project?

9. What would you like to share with other RESET sites?

Prompt: What do you think your hospital is doing well, what makes you proud?

**Conclusion:**

Is there anything else that you would like to tell us about, or make sure that we learn about while we are here today?

Thanks for agreeing to take time to meet with us today . . . .
